# Impact of the COVID-19 Pandemic on Influenza Hospital Admissions and Deaths in Wales: Descriptive National Time Series Analysis

**DOI:** 10.2196/43173

**Published:** 2024-08-21

**Authors:** Mohammad Alsallakh, Davies Adeloye, Eleftheria Vasileiou, Shanya Sivakumaran, Ashley Akbari, Ronan A Lyons, Chris Robertson, Igor Rudan, Gwyneth A Davies, Aziz Sheikh

**Affiliations:** 1 Population Data Science, Faculty of Medicine, Health and Life Science, Swansea University, Swansea, United Kingdom; 2 School of Health and Life Sciences, Teesside University, Middlesbrough, United Kingdom; 3 Usher Institute, The University of Edinburgh, Edinburgh, United Kingdom; 4 Department of Mathematics and Statistics, University of Strathclyde, Glasgow, United Kingdom; 5 Health Protection, Public Health Scotland, Glasgow, United Kingdom; 6 Nuffield Department of Primary Care Health Sciences, University of Oxford, Oxford, United Kingdom

**Keywords:** influenza, hospitalization, mortality, COVID-19 pandemic, nonpharmaceutical interventions, Wales, COVID-19, community health, hospital admission, endemic virus, public health surveillance

## Abstract

**Background:**

The COVID-19 pandemic and the ensuing implementation of control measures caused widespread societal disruption. These disruptions may also have affected community transmission and seasonal circulation patterns of endemic respiratory viruses.

**Objective:**

We aimed to investigate the impact of COVID-19–related disruption on influenza-related emergency hospital admissions and deaths in Wales in the first 2 years of the pandemic.

**Methods:**

A descriptive analysis of influenza activity was conducted using anonymized pathology, hospitalization, and mortality data from the Secure Anonymised Information Linkage Databank in Wales. The annual incidence of emergency hospitalizations and deaths with influenza-specific diagnosis codes between January 1, 2015, and December 31, 2021, was estimated. Case definitions of emergency hospitalization and death required laboratory confirmation with a polymerase chain reaction test. Trends of admissions and deaths were analyzed monthly and yearly. We conducted 2 sensitivity analyses by extending case definitions to include acute respiratory illnesses with a positive influenza test and by limiting admissions to those with influenza as the primary diagnosis. We also examined yearly influenza testing trends to understand changes in testing behavior during the pandemic.

**Results:**

We studied a population of 3,235,883 Welsh residents in 2020 with a median age of 42.5 (IQR 22.9–61.0) years. Influenza testing in Wales increased notably in the last 2 months of 2020, and particularly in 2021 to 39,720 per 100,000 people, compared to the prepandemic levels (1343 in 2019). The percentage of influenza admissions matched to an influenza polymerase chain reaction test increased from 74.8% (1890/2526) in 2019 to 85.2% (98/115) in 2021. However, admissions with a positive test per 100,000 population decreased from 17.0 in 2019 to 2.7 and 0.6 in 2020 and 2021, respectively. Similarly, deaths due to influenza with a positive influenza test per 100,000 population decreased from 0.4 in 2019 to 0.0 in 2020 and 2021. Sensitivity analyses showed similar patterns of decreasing influenza admissions and deaths in the first 2 years of the COVID-19 pandemic.

**Conclusions:**

Nonpharmaceutical interventions to control COVID-19 were associated with a substantial reduction in the transmission of the influenza virus, with associated substantial reductions in hospital cases and deaths observed. Beyond the pandemic context, consideration should be given to the role of nonpharmaceutical community-driven interventions to reduce the burden of influenza.

## Introduction

COVID-19 has resulted in enormous national, regional, and global health and economic and societal impacts, with 24.9 million cumulative cases and over 232,112 deaths in the United Kingdom as of February 4, 2024 [1]. The absence of effective and safe antiviral treatments or vaccines in the early stages of the pandemic [2] led to a variety of nonpharmaceutical interventions (NPIs) worldwide, including social distancing, mask wearing, handwashing, self-isolation, border closure, and lockdowns [3].

A growing body of evidence suggests these NPIs might have also reduced the transmission of other respiratory pathogens, such as the influenza virus, that have similar routes of transmission of SARS-CoV-2. Variable degrees of reduction in the activity of the influenza virus during the COVID-19 pandemic have been reported globally. Evidence from temperate zones of the Southern Hemisphere showed that influenza virus activity during the first wave of the COVID-19 pandemic was lower than historical seasons, despite continued or even increased testing for influenza viruses in some countries [4,5]. Several studies from these regions have attributed the observed reduction of influenza virus activity to the implementation of NPIs for COVID-19, these coinciding with the peak of the influenza season [6-9]. Furthermore, in several African countries, where more school closures have been implemented at various levels during the pandemic, stronger implementation of school closures was associated with a 20% reduction in influenza positivity rate [10].

Surveillance reports in the Northern Hemisphere showed similar patterns of declining levels of influenza activity in the early stages of the COVID-19 pandemic [5], with studies estimating a 64% reduction in incidence in China and Singapore and a 44% reduction in transmissibility in Hong Kong [8,11,12]. In China, NPIs were associated with a substantial reduction in both the number of influenza-like illness cases and infection duration [13]. In South Korea, NPIs, such as school closures, travel restrictions, and face mask use, were associated with a 91%‐100% reduction in influenza incidence [14,15]. Furthermore, the influenza positivity rate declined in the first year of the pandemic in India and Saudi Arabia [16,17]. Similarly, among the UK Armed Forces population, NPIs in the first year of the COVID-19 pandemic have been negatively correlated with the incidence of influenza-like illness [18].

Given the substantial burden of influenza on health care systems and its potential to cause severe illnesses and deaths among high-risk groups, it is important to understand the impact of NPIs enacted during the COVID-19 pandemic on severe influenza outcomes. A few studies have examined this impact by analyzing the impact of these NPIs on influenza-related hospitalization and critical care [7,11,19-21], although data regarding the impact on influenza mortality are limited [22].

In this study, we sought to explore influenza activity in Wales, United Kingdom, during the first 2 years of the COVID-19 pandemic and the preceding 5 years. Specifically, we aimed to investigate the potential impact of the implementation of NPIs during the COVID-19 pandemic on severe influenza illness leading to emergency hospital admission or death.

## Methods

### Ethical Considerations

In this study, we only used fully anonymized data from the Secure Anonymised Information Linkage (SAIL) Databank [23-26]. The SAIL’s independent Information Governance Review Panel approved this study (project 0911). This study was exempted from human subject research ethics review, and the requirement for informed consent was waived because the study only used fully anonymized data.

### Data Sources

We used anonymized population-scale, individual-level linked data from the SAIL Databank in Wales [23-26]. Within SAIL, we used the following Wales-wide data sources for population-level cohorts that have been set up for COVID-19 research [27]. Demographic data were extracted from the Welsh Demographic Service Dataset. We extracted data on laboratory tests for influenza from the Welsh Results Reports Service (WRRS) data set. The WRRS data set contains pathology records with increasing geographical coverage across Wales over the last decade. Hospital admission data were extracted from the Patient Episode Database for Wales, which include secondary care data from all National Health Service hospitals in Wales. We extracted mortality data from the Annual District Death Extract (ADDE) data set, which is based on the Office for National Statistics mortality register, in which cause of death data is regularly checked and validated [28]. All the above data sources were linked together using SAIL’s Anonymised Linkage Field.

### Study Design, Population, and Case Definitions

This study follows a descriptive research design using data about the entire population of Wales. We extracted emergency hospital admissions and deaths due to influenza infection between January 1, 2015, and December 31, 2021. Since our case definitions involve laboratory confirmation, we determined the study’s start date based on our assessment of the quality and coverage of influenza testing data in the WRRS data set at the time of analysis.

We defined an emergency influenza hospital admission based on the following criteria: having influenza (J09-J11) recorded in any diagnosis position using the *International Statistical Classification of Diseases, Tenth Revision* (*ICD-10*); the route to hospital was via emergency department, bed bureau, referral from a general practitioner or consultant for immediate admission, or National Health Service Direct [29]; and a positive result for influenza A or B virus using a polymerase chain reaction (PCR) test, collected within 7 days before or after admission date.

A death due to influenza was defined as a death record in the ADDE data set with an *ICD-10* code of influenza (J09-J11) as the underlying cause of death and a positive result for influenza A or B virus using a PCR test, collected in the last 28 days before death.

Multimedia Appendix 1 contains a list of *ICD-10* codes used to define influenza and influenza-related illness.

### Analysis

We present the incidence of influenza tests and hospital admissions and deaths due to influenza over the period from January 2015 to December 2021, with the denominator being the entire population of Wales. We present the trends of influenza tests per month throughout the study period. We then present the trends of admissions and deaths both per month and per year by the result of the matching influenza test, if any. For each year, we calculated the percentage of influenza admissions and deaths with a matching influenza test including those that satisfied our case definitions, that is, with a positive influenza test.

Two sensitivity analyses were then performed: in the first, we extended the diagnosis code set to include influenza-related respiratory illnesses with acute presentations, in addition to influenza, in line with approaches used elsewhere [30-32]. In the second sensitivity analysis, emergency admissions were limited to those with an influenza-specific code as the primary diagnosis.

The analysis was performed in R (version 4.1.3; R Foundation for Statistical Computing). Records with low-quality linkage or a missing linkage field were excluded from the analysis. Appropriate disclosure control checks were carried out to maintain privacy protection and avoid patient identification in line with SAIL’s information governance policy. These include replacing counts smaller than 5 with 0.

### Reporting

The STROBE (Strengthening the Reporting of Observational Studies in Epidemiology) and the RECORD (Reporting of Studies Conducted Using Observational Routinely-Collected Health Data) statements were followed in the reporting of this study [33,34].

## Results

The study is based on the entire population of Wales. A total of 3,235,883 had a residential address in Wales in mid-2020, the population was 50.7% (1,641,970/3,235,883) female, and the median age was 42.5 (IQR 22.9–61.0) years.

### Influenza Tests

**Figure 1.**
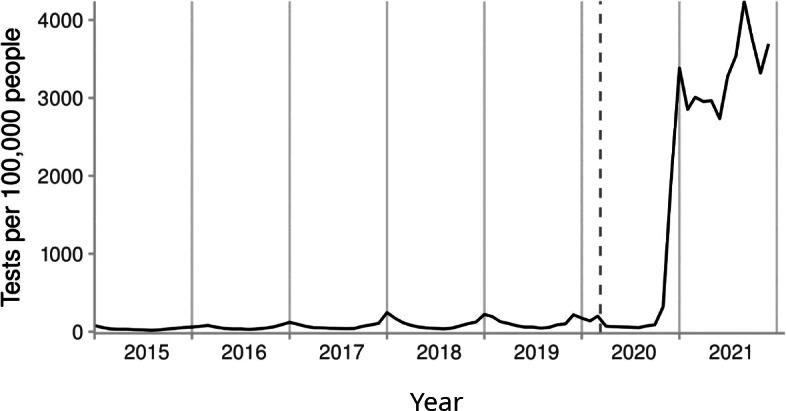
Number of polymerase chain reaction tests for influenza A and B in Wales per month from 2015 to 2021. The dashed vertical line represents the date on which the COVID-19 pandemic was declared. Influenza test records were extracted from the Welsh Results Reports Service data source.

The number of influenza PCR tests per 100,000 people slowly increased from 461.3 in 2015 to 1343 in 2019. However, these tests increased notably in the last 2 months of 2020, raising the total in that year to 3237. In 2021, tests increased substantially to 39,720 per 100,000 people (Figure 1 and Table 1).

**Table 1.** Number of polymerase chain reaction tests for influenza A and B in Wales per year from 2015 to 2021. Influenza test records were extracted from the Welsh Results Reports Service data source.

Year	Population, n	Tests, n	Tests per 100,000 population, n
2015	3,202,489	14,773	461.3
2016	3,210,914	20,890	650.6
2017	3,219,095	26,414	820.5
2018	3,226,596	37,158	1151.6
2019	3,234,939	43,445	1343.0
2020	3,235,883	104,605	3232.7
2021	3,243,287	1,288,234	39,720.0

### Admissions

**Figure 2.**
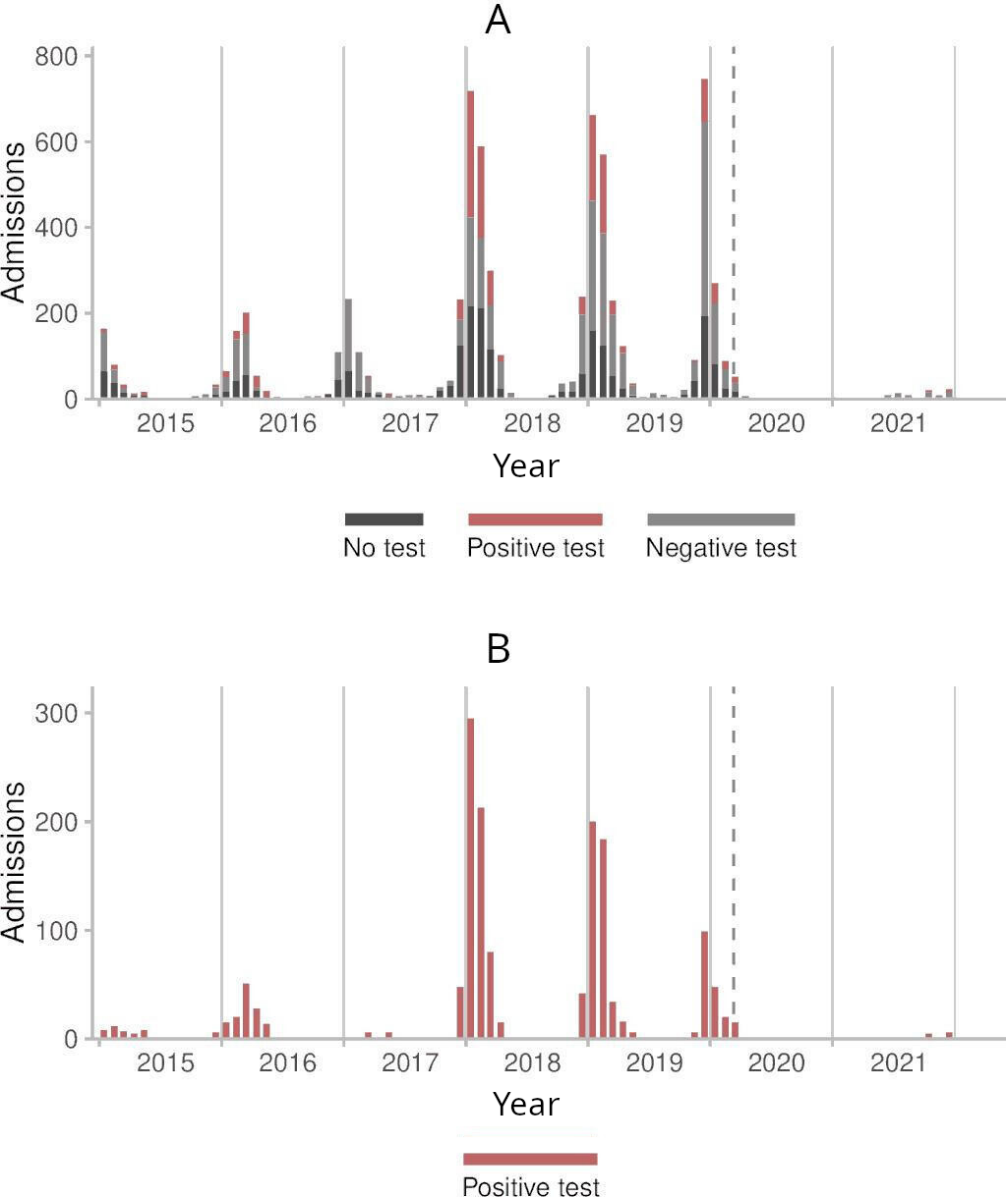
Monthly counts of admissions with influenza-specific codes at any diagnosis position in Wales before and during the COVID-19 pandemic. Counts smaller than 5 are suppressed. (A) Stacked bar chart showing the counts of all admissions per influenza polymerase chain reaction status (no test, positive test, or negative test). (B) Bar chart showing the counts of admissions with positive tests only. The dashed vertical line represents the date on which the COVID-19 pandemic was declared. Admission records were extracted from the Patient Episode Database for Wales. Influenza test records were extracted from the Welsh Results Reports Service data source.

Before the COVID-19 pandemic, the annual incidence rates per 100,000 population of admissions with influenza-specific diagnosis codes ranged from 12.0 in 2015 to 78.1 in 2019. This decreased to 13.5 and 3.5 in 2020 and 2021, respectively (Table 2). The percentage of those admissions that could be matched to an influenza PCR test increased from 56.7% (217/383) in 2015 to 74.8% (1890/2526) in 2019 and reached 85.2% (98/115) in 2021. There were no marked changes in test positivity among those admissions during the pandemic. However, the number of admissions with a positive test per 100,000 people decreased from 17.0 in 2019 to 2.7 and 0.6 in 2020 and 2021, respectively. Figure 2 shows the monthly trend of admissions with influenza-specific diagnosis codes over the study period.

**Table 2.** Annual incidence of emergency admissions with influenza-specific *International Statistical Classification of Diseases, Tenth Revision* (*ICD-10*) codes at any diagnosis position, including those with influenza polymerase chain reaction test.^a^

Year	Population	Admissions with influenza-specific *ICD-10* codes at any diagnosis position			
		Total, n (per 100,000 population)	With influenza test		
			Total, n (%)	Positive, n (per 100,000 population)^b^	Positivity (%)^c^
2015	3,202,489	383 (12.0)	217 (56.7)	46 (1.4)	21.2
2016	3,210,914	652 (20.3)	446 (68.4)	129 (4.0)	28.9
2017	3,219,095	784 (24.4)	465 (59.3)	70 (2.2)	15.1
2018	3,226,596	2078 (64.4)	1395 (67.1)	658 (20.4)	47.2
2019	3,234,939	2526 (78.1)	1890 (74.8)	551 (17.0)	29.2
2020	3,235,883	436 (13.5)	299 (68.6)	87 (2.7)	29.1
2021	3,243,287	115 (3.5)	98 (85.2)	19 (0.6)	19.4

a For each year between 2015 and 2021, the table includes the population estimate for Wales, the total number of admissions with influenza-specific *ICD-10* codes, the number of those admissions that match an influenza test collected within 7 days before or after admission date, the number of those admissions with positive tests, and test positivity. Admission records were extracted from the Patient Episode Database for Wales. Influenza test records were extracted from the Welsh Results Reports Service data source.

b This column represents the case definition: emergency admission with an influenza-specific *ICD-10* code at any diagnosis position and a positive influenza test.

c Positivity % is the percentage of emergency admissions with influenza-specific *ICD-10* codes and an influenza test where the test was positive.

### Deaths

Before the pandemic, the annual incidence rates per 100,000 population of deaths with influenza-specific *ICD-10* codes as the underlying cause ranged from 0.6 (2015) to 2.3 (2018), before decreasing to 0.7 and 0.0 deaths in 2020 and 2021, respectively (Table 3). The percentage of those deaths that could be matched to an influenza PCR test ranged before the pandemic between 72.7% (16/22) in 2016 and 77.3% (58/75) in 2018 before decreasing to 60.9% (14/23) in 2020. The percentage of tests with positive results ranged between 0% and 56.9% (33/58) before the pandemic. With laboratory confirmation of influenza, the incidence rate ranged between 0.0 to 1.0 deaths per 100,000 people before the pandemic, whereas no deaths with positive tests were identified in either 2020 or 2021.

The sensitivity analyses in Multimedia Appendix 2 demonstrate similar trends of decreasing laboratory-confirmed influenza admissions and deaths during the COVID-19 pandemic using the wider case definitions (ie, by extending the diagnosis code set to include influenza-related acute respiratory illnesses) as well as the trends of laboratory-confirmed admissions with a primary diagnosis of influenza.

**Table 3.** Annual incidence of deaths with influenza as the underlying cause, including those with influenza polymerase chain reaction test.^a^

Year	Population	Deaths with influenza as the underlying cause			
		Total, n (per 100,000 population)	With influenza test		
			Total, n (%)	Positive, n (per 100,000 population)^b^	Positivity (%)^c^
2015	3,202,489	20 (0.6)	15 (75)	0 (0)^d^	0
2016	3,210,914	22 (0.7)	16 (72.7)	5 (0.2)	31.3
2017	3,219,095	26 (0.8)	19 (73.1)	0 (0.0)	0
2018	3,226,596	75 (2.3)	58 (77.3)	33 (1.0)	56.9
2019	3,234,939	52 (1.6)	40 (76.9)	12 (0.4)	30
2020	3,235,883	23 (0.7)	14 (60.9)	0 (0)	0
2021	3,243,287	0 (0)	0 (0)	0 (0)	—^e^

a For each year between 2015 and 2021, the table includes the population estimate for Wales, the total number of deaths with influenza as the underlying cause, the number of those deaths that match an influenza test collected in the last 28 days before death, the number of those deaths with positive tests, and test positivity. Death records were extracted from the Annual District Death Extract data set. Influenza test records were extracted from the Welsh Results Reports Service data source.

b This column represents our case definition: death due to influenza as the underlying cause with a positive influenza test.

c Positivity % is the percentage of deaths with influenza as the underlying cause and an influenza test where the test was positive.

d Counts fewer than 5 are reported as 0.

e Not available.

## Discussion

### Principal Findings

This national analysis has demonstrated a substantial reduction in the incidence of influenza and influenza-associated hospitalizations and deaths during the first 2 years of the COVID-19 pandemic compared with prepandemic levels. This reduction was also seen in our sensitivity analyses using wider case definitions of laboratory-confirmed influenza hospitalizations and deaths. We also observed a surge in influenza testing toward the end of 2020 and throughout the second year of the pandemic.

### Interpretation

The significant reductions in influenza admissions and deaths in Wales that we observed during the COVID-19 pandemic are unlikely to be attributed to natural fluctuations in influenza activity over the years. Following the 2009‐2010 H1N1 pandemic, Public Health Wales influenza surveillance reports showed a surge in general practitioner consultations for influenza-like illness, peaking in the 2010‐2011 season, then decreasing to low to medium intensity in the subsequent years. A notable increase occurred in 2017‐2018, followed by a less severe 2018‐2019 season and a shorter 2019‐2020 season [35]. However, the significantly reduced influenza activity during the first 2 years of the COVID-19 pandemic was unlikely to be a mere continuation of the preceding downward trend, and it was even lower than the multiyear low- to medium-intensity activity prior to the 2017‐2018 surge. Instead, it is more plausible that this reduction was mainly due to diminished influenza virus circulation due to the widespread implementation of NPIs. This interpretation is corroborated by the resurgence of influenza activity following the lifting of public health restrictions in early 2022, with the influenza 2022‐2023 season exhibiting levels of activity comparable to those observed in the prepandemic 2019‐2020 season [36]. In the rest of the United Kingdom, broadly similar patterns were observed, as influenza activity was diminished during the period of COVID-19 restrictions and resurged in the 2022‐2023 season after the restrictions were lifted [37]. The reduction in the incidence of influenza during COVID-19–related NPIs can be explained by the similar pathways of infection between the 2 viral diseases [8].

In addition to the impact of COVID-19–related NPIs, influenza immunization might have contributed to the reduction we observed in severe influenza outcomes. Public Health Wales has reported that influenza vaccine uptake rates in people 65 years or older in Wales have increased from 65.7% and 67.1% in 2018/2019 and 2019/2020 to 76.5% in 2020/2021 [38].

The decline in influenza activity during the pandemic is unlikely due to underdetection, given that we observed a substantial rise in influenza testing during the 2020‐2021 winter season compared to nonpandemic years. This surge in testing could be largely explained by the use of respiratory multiplex testing and serious concerns about a possible concurrent rise in influenza and COVID-19 cases and the potential severity of coinfection [39].

There is a significant overlap between the population groups susceptible to severe illness from influenza and COVID-19 [40,41]. It is possible that high COVID-19 mortality has led to a reduction in the influenza-susceptible population, particularly older people and people with chronic health conditions, which might have contributed to the reduction in influenza severe outcomes during the pandemic.

### Comparison With Prior Work

Several reports have suggested that the pandemic has had direct effects on the circulation of influenza virus in the population. Koutsakos et al [42] noted a considerable global reduction in influenza cases for both influenza A and B viruses in their study. They also reported a globally disappearing influenza *B/Yamagata* lineage, suggesting that the lineage may have become extinct during the pandemic. A later analysis of the World Health Organization-FluNet database confirmed low levels of *B/Yamagata* lineage being detected during the COVID-19 pandemic up to March 2023 [43]. It may also be argued that the *B/Yamagata* lineage may have already been at a low prevalence at the beginning of the pandemic due to the previous history of its inherent volatility, lower reproductive strength, slower growth phase, and shorter transmission chains [42]. The rapid decline of the *B/Yamagata* lineage throughout the COVID-19 pandemic has made experts suggest a more pronounced vulnerability and breakdown of the lineage, especially with the widespread movement restrictions and social distancing conditions [42,44].

In a systematic review of studies from 15 countries, Fricke et al [45] further suggested that NPIs targeted at SARS-CoV-2 transmission reduced influenza burden in many settings, with recommendations for low-threshold NPIs in influenza prevention programs. In South Korea, NPIs were widely implemented to mitigate the spread of COVID-19, with a decrease in hospitalizations for influenza, pneumonia, asthma, and chronic obstructive pulmonary disease reported during the intervention [46]. In China, also with strict NPIs aimed at controlling COVID-19, almost a two-thirds reduction in the incidence rate of seasonal influenza was reported in 2020 [8,47]. Another study analyzed ED visits, hospitalizations, and ICU admissions in 4 hospitals in France during the 2020 influenza season, the first wave of COVID-19, and lockdown period [48] and found a surge in influenza emergency department visits that heralded the influenza season, followed by a mild decline in cases during the first wave, and thereafter by a significant decrease during and after lockdown. As observed in this study, these reports all further align with findings in our previous studies in Scotland and Wales [49,50]. We reported that UK-wide lockdowns were associated with about 40% and 50% reductions in severe asthma and chronic obstructive pulmonary disease exacerbations, respectively, across both Scotland and Wales, with no corresponding increase in deaths [49,50].

### Implications for Public Health Policy

Influenza has been a major cause of morbidity and mortality as well as a significant economic burden in the United Kingdom and globally [51-53]. Our findings indicate that the widespread implementation of NPIs has also impacted influenza circulation, which is consistent with global data. This suggests that, beyond the COVID-19 context, strategic NPIs used at scale could potentially reduce influenza incidence and improve outcomes.

While strict measures, such as lockdowns, are most unlikely to be politically or socially acceptable nor feasible in the long term, other NPIs, such as regular handwashing, wearing of face masks where needed, and reducing social contact or even self-isolation when potentially infected, may be more acceptable, particularly if focused on and around the main influenza season. In addition to NPIs, vaccination is one of the most effective preventive measures against influenza, and there is considerable merit in drives to promote and maintain high levels of influenza vaccine uptake, as this was certainly suboptimal in Wales [54]. The aforementioned measures should be coupled with innovative, comprehensive public health education strategies, such as ground-up, community-driven approaches, to increase public awareness about the importance of these preventive measures, how to correctly implement them, and to challenge misconceptions about influenza [55,56]. However, the feasibility, acceptability, and effectiveness of NPIs can vary significantly between countries due to factors, such as population density, political systems, and sociocultural factors [47,57-59].

The potential impact of COVID-19–related NPIs on influenza perhaps extends to other common respiratory infections [8,46]. For example, Song et al [60] clearly noted in a 2020 study of about 75,000 children in the United States that COVID-19 public health interventions were highly effective in preventing seasonal respiratory viral infections, such as respiratory syncytial virus, human parainfluenza virus, and human metapneumovirus, besides influenza. A decline in circulation of other respiratory pathogens, such as rhinovirus, respiratory enterovirus, and *Streptococcus pneumoniae*, have been also reported at the onset of the pandemic, when the strictest NPIs were implemented, before rebounding to varying degrees later [22].

Careful consideration should be given to a likely interaction between the influenza virus and SARS-CoV-2, especially when the winter approaches or in the event of another viral respiratory outbreak. Studies have suggested that coinfection could have a significant impact on disease burden and mortality. Stowe et al [61] noted that coinfection with the influenza virus had about 6 times higher risk of death than those with neither influenza nor SARS-CoV-2, whereas Swets et al [62] reported that coinfection with the influenza virus increased the probability of invasive mechanical ventilation by about 4 times and doubled in-hospital mortality.

The significant decrease in exposure to influenza during the COVID-19 pandemic could potentially diminish population immunity and increase the severity of future influenza epidemics [43]. Therefore, it is important to closely monitor influenza activity and its circulating subtypes and strengthen vaccine uptake.

### Limitations

Unlike our previous studies [49,50], we did not directly model the impact of NPIs on influenza admissions and deaths. Rather, we descriptively analyzed the available data and presented our estimates as such, which have implications for statistical inference and study interpretations. In addition, we did not evaluate the potential effect of influenza vaccination on hospitalizations and deaths. Our analysis did not account for influenza strains or weather factors, which might have influenced the changes in influenza circulation patterns and associated hospitalizations and deaths during the pandemic. We also did not examine whether the incidence of influenza severe illness might have been affected by COVID-19–related mortality displacement or a potential reduction of influenza-vulnerable population due to high COVID-19 mortality. Therefore, we are unable to infer a causal relationship between NPIs and influenza emergency admissions and deaths. However, this causal relationship is highly plausible, and our findings, based on 5-year prepandemic and 2-year pandemic nationwide data, are consistent with an abundance of evidence from around the world that NPIs targeted at SARS-CoV-2 transmission might have also reduced influenza burden as well.

Our case definitions of influenza admissions and deaths prioritized specificity over sensitivity by requiring influenza-specific *ICD-10* codes besides laboratory confirmation. Influenza-specific diagnosis codes have been used elsewhere to identify influenza hospitalizations [63,64]. However, although these diagnosis codes have been shown to have high specificity to identify laboratory-confirmed influenza hospitalizations, they have only moderate sensitivity [65-68]. In addition, the version of the WRRS data set from which we extracted influenza testing data might have incomplete geographical coverage. Nonetheless, we are more interested in trends than counts, and our sensitivity analysis has indeed demonstrated similar trends of laboratory-confirmed influenza hospitalizations and deaths before and during the COVID-19 pandemic using liberal case definitions. In these case definitions, we extended the diagnosis code set to include a wide range of non–COVID-19 acute respiratory illnesses. This ensured the capture of a broader range of admissions and deaths due to influenza and influenza-related exacerbation of respiratory conditions, including cases that lacked an influenza diagnosis code. Since an influenza swab is often collected when there is a reasonable clinical suspicion of influenza, laboratory confirmation of influenza controls for the wide list of acute respiratory illnesses and keeps the case definition reasonably specific to influenza. This liberal approach has been adopted by the World Health Organization and other studies [30,32,65,69,70].

### Conclusions

Our national, population-based data show substantial reductions in influenza-associated hospitalization and mortality in Wales over the first 2 years of the pandemic compared with prepandemic levels. These reductions are unlikely to reflect changes in testing behavior. Rather, these most likely reflect a decrease in circulation and exposure to the influenza virus associated with the widespread adoption of a range of NPIs targeted at COVID-19.

The abundant evidence about the impact of large-scale NPIs on influenza could redefine our approach to influenza control in nonpandemic contexts. A comprehensive approach to influenza control that strategically incorporates NPIs, innovative public health education approaches, and effective vaccination programs could potentially lead to sustained reductions in influenza incidence and associated morbidity and mortality. Given the likely challenges of implementing NPIs outside the pandemic context, research and policy should focus on understanding the barriers to their implementation and piloting proactive, community-led public health strategies to reduce the burden of influenza.

## Supplementary material

10.2196/43173 Multimedia Appendix 1 The *International Statistical Classification of Diseases, Tenth Revision* (*ICD-10*) code set.

10.2196/43173 Multimedia Appendix 2 Sensitivity analyses.

## Abbreviations

ADDE: Annual District Death Extract
*ICD-10*: *International Statistical Classification of Diseases, Tenth Revision*
NPI: nonpharmaceutical intervention
PCR: polymerase chain reaction
RECORD: Reporting of Studies Conducted Using Observational Routinely-Collected Health Data
SAIL: Secure Anonymised Information Linkage
STROBE: Strengthening the Reporting of Observational Studies in Epidemiology
WRRS: Welsh Results Reports Service
