# Supplementary material for: Impact of the COVID-19 Pandemic on Influenza Hospital Admissions and Deaths in Wales: Descriptive National Time Series Analysis
Source: JMIR Public Health Surveill. 2024 Aug 21;10:e43173. doi: 10.2196/43173 (PMC11358661; doi:10.2196/43173)
Supplement: Multimedia Appendix 1 [file publichealth-v10-e43173-s001.pdf]

Multimedia Appendix 1: Code set

ICD-10 codes for influenza and influenza-related illness

Group	ICD-10 code	Description
Influenza codes		
Influenza	J09	Influenza due to identified zoonotic or pandemic influenza virus
	J10	Influenza due to identified seasonal influenza virus
	J10.0	Influenza with pneumonia, seasonal influenza virus identified
	J10.1	Influenza with other respiratory manifestations, seasonal influenza virus identified
	J10.8	Influenza with other manifestations, seasonal influenza virus identified
	J11	Influenza, virus not identified
	J11.0	Influenza with pneumonia, virus not identified
	J11.1	Influenza with other respiratory manifestations, virus not identified
	J11.8	Influenza with other manifestations, virus not identified
Influenza-related illness codes		
Other pneumonia	J12	Viral pneumonia, not elsewhere classified
	J12.0	Adenoviral pneumonia
	J12.1	Respiratory syncytial virus pneumonia
	J12.2	Parainfluenza virus pneumonia
	J12.3	Human metapneumovirus pneumonia
	J12.8	Other viral pneumonia
	J12.9	Viral pneumonia, unspecified
	J13	Pneumonia due to Streptococcus pneumoniae
	J14	Pneumonia due to Haemophilus influenzae
	J15	Bacterial pneumonia, not elsewhere classified
	J15.0	Pneumonia due to Klebsiella pneumoniae
	J15.1	Pneumonia due to Pseudomonas
	J15.2	Pneumonia due to staphylococcus
	J15.3	Pneumonia due to streptococcus, group B
	J15.4	Pneumonia due to other streptococci
	J15.5	Pneumonia due to Escherichia coli
	J15.6	Pneumonia due to other Gram-negative bacteria
	J15.7	Pneumonia due to Mycoplasma pneumoniae
	J15.8	Other bacterial pneumonia
	J15.9	Bacterial pneumonia, unspecified
	J16	Pneumonia due to other infectious organisms, not elsewhere classified
	J16.0	Chlamydial pneumonia
	J16.8	Pneumonia due to other specified infectious organisms
	J17	Pneumonia in diseases classified elsewhere
	J17.0	Pneumonia in bacterial diseases classified elsewhere
	J17.1	Pneumonia in viral diseases classified elsewhere
	J17.2	Pneumonia in mycoses
	J17.3	Pneumonia in parasitic diseases
	J17.8	Pneumonia in other diseases classified elsewhere
	J18	Pneumonia, organism unspecified
	J18.0	Bronchopneumonia, unspecified
	J18.1	Lobar pneumonia, unspecified
	J18.2	Hypostatic pneumonia, unspecified
	J18.8	Other pneumonia, organism unspecified
	J18.9	Pneumonia, unspecified
Other Lower Respiratory Tract Infection (LRTI)	J20	Acute bronchitis
	J20.0	Acute bronchitis due to Mycoplasma pneumoniae
	J20.1	Acute bronchitis due to Haemophilus influenzae
	J20.2	Acute bronchitis due to streptococcus

	J20.3	Acute bronchitis due to coxsackievirus
	J20.4	Acute bronchitis due to parainfluenza virus
	J20.5	Acute bronchitis due to respiratory syncytial virus
	J20.6	Acute bronchitis due to rhinovirus
	J20.7	Acute bronchitis due to echovirus
	J20.8	Acute bronchitis due to other specified organisms
	J20.9	Acute bronchitis, unspecified
	J21	Acute bronchiolitis
	J21.0	Acute bronchiolitis due to respiratory syncytial virus
	J21.1	Acute bronchiolitis due to human metapneumovirus
	J21.8	Acute bronchiolitis due to other specified organisms
	J21.9	Acute bronchiolitis, unspecified
	J22	Unspecified acute lower respiratory infection
Chronic lower respiratory diseases (bronchitis, COPD, asthma, bronchiectasis)	J40	Bronchitis, not specified as acute or chronic
	J41	Simple and mucopurulent chronic bronchitis
	J41.0	Simple chronic bronchitis
	J41.1	Mucopurulent chronic bronchitis
	J41.8	Mixed simple and mucopurulent chronic bronchitis
	J42	Unspecified chronic bronchitis
	J43	Emphysema
	J43.0	MacLeod syndrome
	J43.1	Panlobular emphysema
	J43.2	Centrilobular emphysema
	J43.8	Other emphysema
	J43.9	Emphysema, unspecified
	J44	Other chronic obstructive pulmonary disease
	J44.0	Chronic obstructive pulmonary disease with acute lower respiratory infection
	J44.1	Chronic obstructive pulmonary disease with acute exacerbation, unspecified
	J44.8	Other specified chronic obstructive pulmonary disease
	J44.9	Chronic obstructive pulmonary disease, unspecified
	J45	Asthma
	J45.0	Predominantly allergic asthma
	J45.1	Nonallergic asthma
	J45.8	Mixed asthma
	J45.9	Asthma, unspecified
	J46	Status asthmaticus
	J47	Bronchiectasis
Viral infection	B34.9	Viral infection, unspecified
Respiratory/ chest symptoms	R05	Cough
	R06.0	Dyspnoea
	R07	Pain in throat and chest
	R06.8	Other and unspecified abnormalities of breathing
	R07.0	Pain in throat
	R07.1	Chest pain on breathing
	R07.2	Precordial pain
	R07.3	Other chest pain
	R07.4	Chest pain, unspecified
	R13	Dysphagia
Generalised symptoms	R50.9	Fever, unspecified
	R51	Headache
	M79.1	Myalgia
	R53	Malaise and fatigue
	F05	Infective delirium
Systemic Inflammatory Response Syndrome (SIRS)	R65.0	Systemic Inflammatory Response Syndrome of infectious origin without organ failure
	R65.1	Systemic Inflammatory Response Syndrome of infectious origin with organ failure
Other	A08.4	Viral intestinal infection, unspecified
