# Supplementary material for: Impact of the COVID-19 Pandemic on Influenza Hospital Admissions and Deaths in Wales: Descriptive National Time Series Analysis
Source: JMIR Public Health Surveill. 2024 Aug 21;10:e43173. doi: 10.2196/43173 (PMC11358661; doi:10.2196/43173)
Supplement: Multimedia Appendix 2 [file publichealth-v10-e43173-s002.pdf]

Multimedia Appendix 2: Sensitivity analyses

Influenza admissions defined using an ICD-10 code of influenza or a related respiratory illness and a positive influenza RT-PCR test:

Monthly count of admissions due to influenza and related illnesses in Wales before and during the COVID-19 pandemic. Counts smaller than 5 are suppressed. (a) Stacked bar chart showing the counts of all admissions per influenza test status (no test, positive test, or negative test). (b) Bar chart showing the counts of admissions with positive tests only. The dashed vertical line represents the date on which the COVID-19 pandemic was declared. Admission records were extracted from the Patient Episode Database for Wales. Influenza test records were extracted from the Welsh Results Reports Service data source.

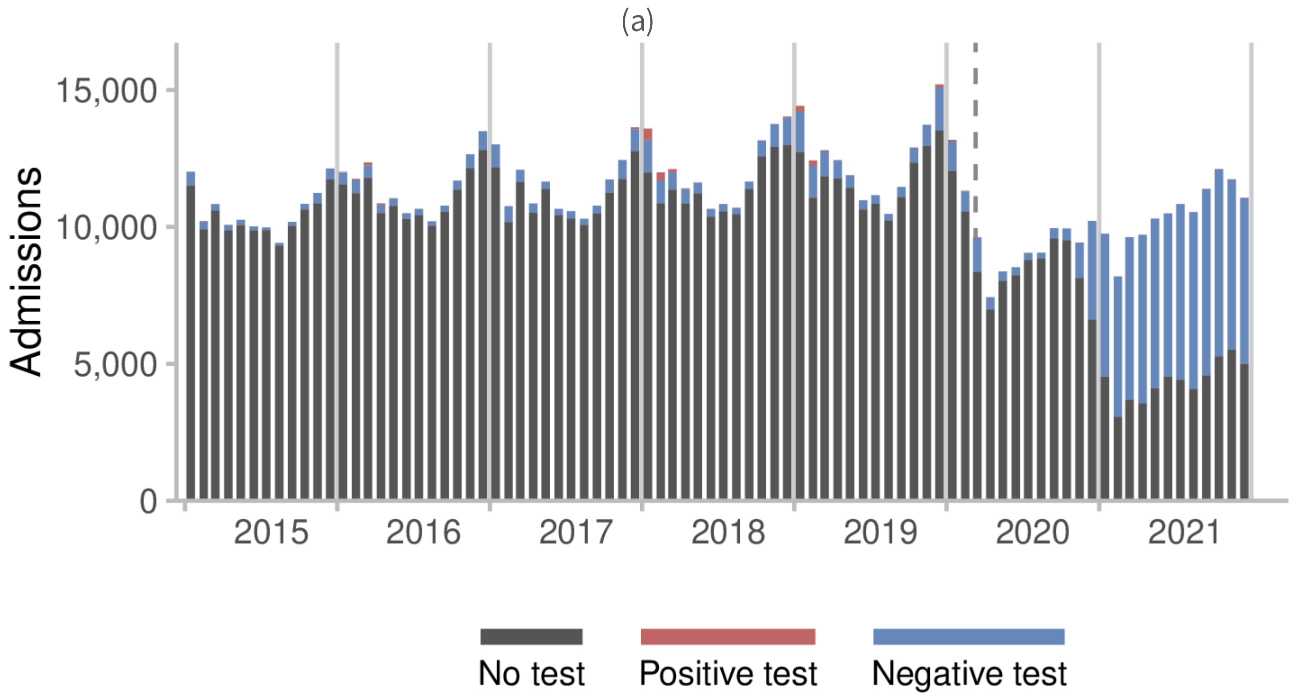

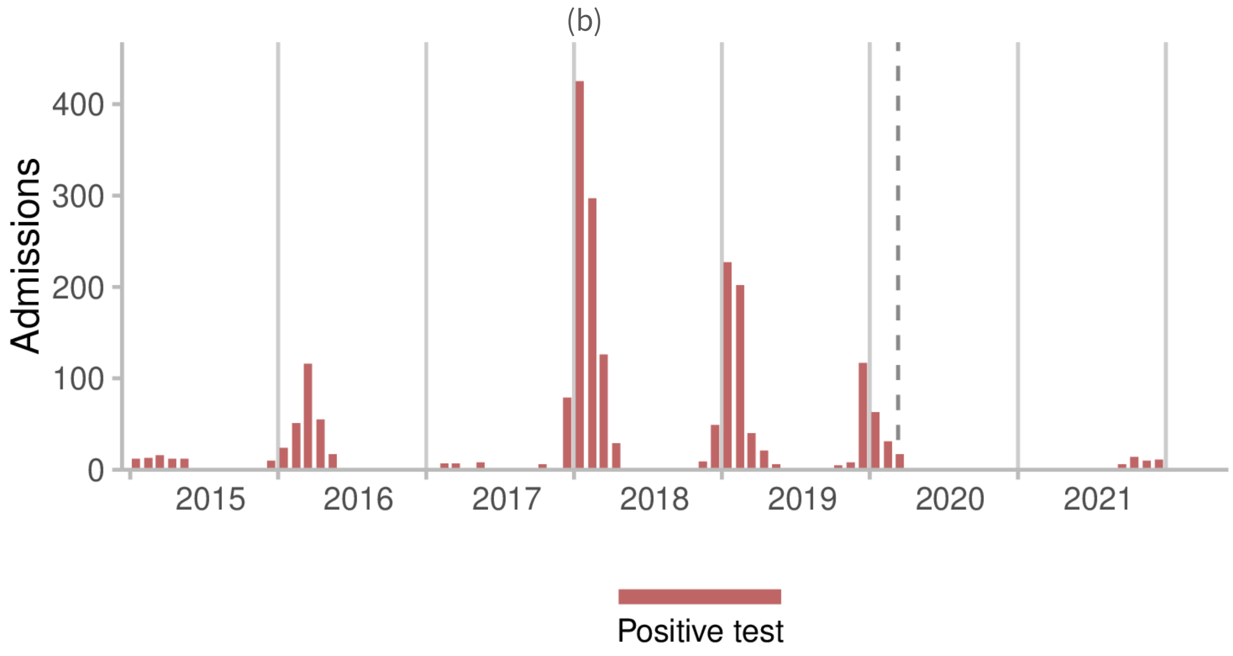

Annual incidence of emergency admissions due to influenza and related respiratory illnesses at any diagnosis position, including those with influenza polymerase chain reaction test. For each year between 2015 and 2021, the table includes the population estimate for Wales, total number of admissions with an ICD-10 code of influenza or a related respiratory illness at any diagnosis position, the number of those admissions that match an influenza test collected within 7 days before or after admission date, the number of those admissions with positive tests, and test positivity. The highlighted column represents the case definition for this sensitivity analysis: emergency admission with an ICD-10 code of influenza or a related respiratory illness at any diagnosis position and a positive influenza test. Admission records were extracted from the Patient Episode Database for Wales. Influenza test records were extracted from the Welsh Results Reports Service data source.

		Admissions with ICD-10 codes of influenza or related respiratory illnesses at any diagnosis position			
		Total	With influenza test		
Year	Population	N (per 100,000 population)	N (%)	Positive (per 100,000 population)	Positivity %[a]
2015	3,202,489	127,216 (3,972.4)	2,930 (2.3)	80 (2.5)	2.7
2016	3,210,914	138,001 (4,297.9)	4,529 (3.3)	267 (8.3)	5.9
2017	3,219,095	138,486 (4,302.0)	5,543 (4.0)	118 (3.7)	2.1
2018	3,226,596	145,510 (4,509.7)	7,938 (5.5)	947 (29.3)	11.9
2019	3,234,939	149,865 (4,632.7)	9,411 (6.3)	629 (19.4)	6.7
2020	3,235,883	116,075 (3,587.1)	10,431 (9.0)	118 (3.6)	1.1
2021	3,243,287	125,723 (3,876.4)	73,395 (58.4)	46 (1.4)	0.1

[a] Positivity % is the percentage of emergency admissions with ICD-10 codes of influenza or related respiratory illnesses and an influenza test where the test was positive.

Influenza admissions defined using an ICD-10 code of influenza as the primary diagnosis and a positive influenza RT-PCR test:

Annual incidence of admissions with influenza-specific ICD-10 codes as the primary diagnosis, including those with influenza polymerase chain reaction test. For each year between 2015 and 2021, the table includes the population estimate for Wales, total number of admissions with an ICD-10 code of influenza or a related respiratory illness as the primary diagnosis, the number of those admissions that match an influenza test collected within 7 days before or after admission date, the number of those admissions with positive tests, and test positivity. The highlighted column represents the case definition for this sensitivity analysis: emergency admission with an ICD-10 code of influenza as the primary diagnosis and a positive influenza test. Admission records were extracted from the Patient Episode Database for Wales. Influenza test records were extracted from the Welsh Results Reports Service data source.

		Admissions with influenza-specific ICD-10 codes as the primary diagnosis			
		Total	With influenza test		
**Year**	**Population**	**N (per 100,000 population)**	**N (%)**	**Positive (per 100,000 population)**	**Positivity %[a]**
2015	3,202,489	252 (7.9)	151 (59.9)	34 (1.1)	22.5
2016	3,210,914	444 (13.8)	318 (71.6)	93 (2.9)	29.2
2017	3,219,095	433 (13.5)	302 (69.7)	51 (1.6)	16.9
2018	3,226,596	1,322 (41.0)	960 (72.6)	459 (14.2)	47.8
2019	3,234,939	1,951 (60.3)	1,543 (79.1)	493 (15.2)	32.0
2020	3,235,883	348 (10.8)	246 (70.7)	65 (2.0)	26.4
2021	3,243,287	56 (1.7)	47 (83.9)	0 (0.0)	0.0

[a] Positivity % is the percentage of emergency admissions with ICD-10 codes of influenza or related respiratory illnesses and an influenza test where the test was positive.

Influenza deaths defined using an ICD-10 code of influenza or a related respiratory illness and a positive influenza RT-PCR test:

Annual incidence of deaths with influenza or a related respiratory illness as the underlying cause, including those with influenza polymerase chain reaction test. For each year between 2015 and 2021, the table includes the population estimate for Wales, total number of deaths with influenza or a related respiratory illness as the underlying cause, the number of those deaths that match an influenza test collected in the last 28 days before death, the number of those deaths with positive tests, and test positivity. The highlighted column represents the case definition for this sensitivity analysis: death with influenza or a related respiratory illness as the underlying cause with a positive influenza test. Death records were extracted from the Annual District Death Extract data source. Influenza test records were extracted from the Welsh Results Reports Service data source.

		Deaths with influenza or a related illness as the underlying cause			
		Total	With influenza test		
Year	Population	N (per 100,000 population)	N (%)	Positive (per 100,00 population)	Positivity %
2015	3,202,489	4,582 (143.1)	160 (3.5)	6 (0.2)	3.8
2016	3,210,914	4,382 (136.5)	214 (4.9)	11 (0.3)	5.1
2017	3,219,095	4,388 (136.3)	292 (6.7)	0 (0.0)	0.0
2018	3,226,596	4,587 (142.2)	313 (6.8)	64 (2.0)	20.4
2019	3,234,939	4,221 (130.5)	285 (6.8)	22 (0.7)	7.7
2020	3,235,883	3,452 (106.7)	301 (8.7)	0 (0.0) [b]	0.0
2021	3,243,287	3,086 (95.2)	1,560 (50.6)	0 (0.0) [b]	0.0

[a] Positivity % is the percentage of deaths with influenza or a related respiratory illness as the underlying cause and an influenza test where the test was positive.

[b] Counts with less than 5 are presented as 0.
